# Evolution in Dentistry

**Published:** 1890-03

**Authors:** J. A. Robinson


### Evolution in Dentistry.

BY J. A. ROBINSON.

Effort is evolution: as when the chick pecks the shell of the egg that it may have life, and a higher and better environment. Everything in life corresponds with every other thing—there is a oneness running through all nature, and science is the key that unlocks all the doors in the great mansion of human life. The turnkey in dentistry for extracting teeth corresponds to the great and terrible despotic rule of the ages that have passed away. Everybody who disobeys the mandate of the king must be destroyed; so every offending tooth must be torn out, and cast into the fire and be burned. The present transition state is proclaiming the supremacy of man over nature. Formerly the heroic practice prevailed in everything; standing armies and brute force ruled everywhere, and offending teeth shared the same despotic fate of all the world. Think of the great step from destroying every offending member, to saving every offending member. Evolution is a plant of slow growth, and the reason progress is so retarded is because our affections are not conjoined to our reflective faculties. Many persons have entered into dentistry more from the love of *ease* than of the love of *use.* Our selfishness overcomes our better nature, and we care more for ourselves than our neighbors; we want to get something for almost nothing. As I had written before, dentistry begun in some mechanical contrivance to arrest the decay and save the teeth, and supply artificial substitutes for teeth that were lost. Gold, tin, and lead were the first and only materials known in Boston fifty-five or sixty years ago; the gold and tin were cut in strips or ribbons and packed around the borders of the cavity making it smaller and smaller until it came to a common center when the end was pushed into the small center hole to retain the whole structure. Now the Herbst method is the same method applied by the engine and corresponds to the improved method of the old plan that was true; the circular saw is the same principle over the hand saw, and the sewing machine the same over sewing by hand. Sewing by machine is a simple knot applied with power, and for all we know, the same knot was tied by mother Eve the morning she tried on her first garment.

The instrument in filling teeth that carried the gold at first was a smooth, blunt-pointed burnisher with a round point. Joshua Tucker introduced the pellets by crimping the gold after it was cut into strips three-fourths of an inch wide between the points of the gold foil carriers, tweezers they were called at that time, the same the watchmaker used in repairing watches. As far as I know I was the first to suggest sharpening the plugger on the sides and end so it would carry the gold into place and not pass through the pellet. Bemis and Flag filled with ribbons when I begun, but Harwood and Tucker filled teeth with soft foil made into pellets.

In those days we treated the pulps of teeth on the same plan we did offending teeth; we cut off the crowns of the six front teeth and extracted the pulp with a pin that was filed three or four sided, with the edges barbed, and thrust it into the canal, gave it a sudden twist, and endeavored to draw the nerve out with one trial; if we did not succeed we tried again. In those days we did not set crowns on any back teeth. The fillings were finished with a strip of sandpaper and a burnisher. I think I was the first person who introduced an instrument for carrying the gold into position that was sharpened on an oil stone to a cutting edge on four sides and the end. I called it a *graver point*, as the sharp sides and the end caught and pushed the gold before the instrument and did not pass through it. I had learned to engrave while in a watch shop; and engraving always carried the part that was removed, before the instrument. The serrated plugger is the same principle extended, as the sandpaper disk is the same thing with which we began, only applied with the power and speed of the dental engine. It is the improved method of the old plan that was true—it was evolution.

About the year 1838 Dr. Mann, an obscure dentist, introduced amalgam under the name of Succedanium Cement, for filling teeth in Boston, and set all the vicinity in a blaze of enquiry what the new material could be that could be put into a tooth in a plastic state and would grow hard in a few hours, and thus prevent further decay; besides he could patch up old, very badly decayed teeth and make them as good as sound ones. About the same time Spooner came out with his recipe of arsenic and morphine, equal parts, to destroy nerves, and the two combined was to make the future of dentistry hopeful and profitable. This took dentistry out of the hands of the M.D's exclusively, and carpenters, and blacksmiths, and printers, and all sorts of people entered into the business of saving teeth. The newspapeis were full of advertisements and circulars of the new discovery, and were sent all over New England. Some one has compared evolution to the branches of a tree growing in all directions; some branches following an upward symmetrical course while others turned in an angular direction, and still other branches retrograding by growing downward ; and it is so in all the callings in human life. Amalgam fillings and extracting teeth without pain have been the retrograding movements in our profession because it is a temptation to do wrong; amalgam fillings are promises that are always broken.

I think a better comparison is to compare amalgam to softwood trees, basswood or linden, the softest wood known and that decays the most rapidly. I saw a farmer once making fence of basswood ; upon asking him why he made fence out of that stuff he replied that "he knew it was good for nothing, but he had no better, and before this rotted out he should be able to get better, or else be so poor that he would not want any," and that is a fit comparison to persons who have teeth filled with amalgam. Since I began writing this I have removed two amalgam fillings that were put in less than three years ago; both teeth were aching and so far gone that their usefulness was almost destroyed. Some of the readers may ask what I filled them with, and I will give the answer further along in evolution. There is but one straight and narrow way in evolution, and amalgam in all its forms is retrogression. If some of my readers reply that they have seen teeth that had been filled with amalgam that lasted a dozen years, I will point them to an equal number of cases that have been decayed as long, and have stood, that have never been filled at all. When Spooner first recommended arsenic and morphine the plan was to give what would now be considered *heroic doses*, and the pulp was so thoroughly poisoned that it was silenced and not removed at all. I have seen cases where the dentine and enamel was colored to a redish-green that run through the whole crowns of large strong teeth; and arsenic was the common practice as much as mercury in the practice in medicine; every one used it; it was *par excellence* and we knew no other way. It oftentimes takes a long while to find out our mistakes, and that what we are doing is good for nothing, and is a failure.

The M.D's, and those who had been educated, were a little more careful in the use of arsenic, but those who had *picked up* dentistry did a great deal of mischief that was only cured by the Wells' discovery of nitrous oxide gas so that they could be removed without pain. The next step in evolution, for evolution only goes forward and upward, was trying to cap the pulps that were exposed with a hollow metallic cap, fitted to a bridge over the opening of the exposed pulp, and fill under the cap with creosote for protection and quiet, and fill with soft-foil or tin; the cap being conical kept off all pressure, and the creosote soothed and quieted the pain and reduced the inflammation and thus saved the tooth. I occasionally read in the dental journals of to-day articles recommending creosote in the treatment of exposed pulps, but it always seems as though the advocates might give better advice by saying carbolic acid in its place; but it only proves the law of evolution, that when the dominant part *declines* it does not perish but takes a subordinate position and lives.

Professors Taft and Watt will perhaps recollect that more than twenty-five years ago I was called at a meeting at Columbus "Old Carbolic Acid," from my enthusiasm in stating what it would do in treating and restoring exposed pulps. In writing strongly against the common use of amalgam I do not wish to be misunderstood, for after carefully examining its merits, and testing every kind I could hear of in the market, and making a good deal myself from various formulæ I could obtain, I became satisfied that the first that was introduced which was made by filing up an old-fashioned Spanish twenty-five cent piece that contained a large per cent. of copper (if we except copper amalgam) and also an experience of more than fifty years of examination of its merits and demerits in saving teeth, I am justified in saying that it is a failure, notwithstanding it has many advocates of its use. I do not wish my sentiments to become vitiated by the *love of ease* when we are over-worked, or of *money-getting* when we are poor; but to intensify, if I can, this hatred to a bad practice that retrogrades the profession, and to stimulate the workers to find some substitute for its use that will benefit the world as well as the dentist. What we need is not what will sometimes be successful in exceptionally strong teeth under favorable circumstances, but something that will save teeth that are friable and of poor material, and tide over a critical period in life, and give strength to teeth, say from six to twenty years of age, and give nature a chance to recuperate and grow strong.

